# Ultrasound and MRI Are Not Interchangeable for Measuring Muscle Cross‐Sectional Area: Analysis of Reliability and Validity in 10 Lower Limb Muscles Considering Sex Differences

**DOI:** 10.1002/ejsc.70145

**Published:** 2026-02-21

**Authors:** Yoko Kunimasa, Robin Macchi, Caroline Nicol, Baptiste Corcelle, Constance P. Michel, Marc‐Adrien Hostin, Lucas Soustelle, David Bendahan

**Affiliations:** ^1^ CNRS ISM‐UMR 7287 Aix‐Marseille University Marseille France; ^2^ Faculty of Education Niigata University Niigata Japan; ^3^ Faculty of Engineering Niigata University Niigata Japan; ^4^ Jean Monnet University Saint‐Étienne France; ^5^ LAMHESS (UPR 6312) University Côte d’Azur Nice France; ^6^ CNRS CRMBM‐UMR 7339 Aix‐Marseille University Marseille France

**Keywords:** extended‐field‐of‐view, lower limb, MRI, panoramic imaging, sex, ultrasonography

## Abstract

Ultrasound‐based anatomical cross‐sectional area (US‐ACSA) measurement using extended‐field‐of‐view (EFOV) imaging offers a practical alternative to magnetic resonance imaging (MRI‐ASCA) measurements. Although its reliability and validity have previously been assessed, previous studies have focused only on a single or limited number of muscles with little attention to effects of muscle size and sex. This study was conducted in ten lower limb muscles of varying sizes in males and females in order to assess the reliability and validity of US‐ACSA measurements using MRI as the gold standard method. Twelve males and twelve females participated in this study. US‐ and MRI‐ACSA were measured twice, one day apart. Reliability was assessed using intraclass correlation coefficients (ICC_2,3_), standard error of measurement (SEM) and minimal detectable change (MDC). Validity was examined using Pearson product‐moment correlation and Bland–Altman analysis. A linear model was used to evaluate the effects of muscle size and sex. ICC_2,3_, SEM and MDC ranged from 0.992% to 0.999%, 0.50%–1.90% and 1.25%–5.36% for US‐ACSA and from 0.952% to 0.998%, 0.90%–4.76% and 2.51%–13.19% for MRI‐ACSA. US‐ACSA showed strong correlations with MRI‐ACSA (*r* = 0.73–0.95). A systematic underestimation of MRI values (relative bias: −11.5% to −23.7%) was identified, with no systematic effect of muscle size or sex. These findings suggest that US‐ACSA measurements of lower limb muscles are highly reliable regardless of muscle size and sex. However, due to the large systematic bias, US‐ and MRI‐based ACSAs measurements are not interchangeable.

## Highlights

- Ultrasound‐based ACSA measurements using the extended‐field‐of‐view method are highly reliable for a wide range of lower limb muscles, regardless of muscle size or participant sex.
- Ultrasound‐ and MRI‐based ACSA measurements were strongly correlated for most muscles; however, ultrasound systematically underestimated ACSA values.
- Findings suggest that, regardless of muscle size or sex, ultrasound‐ and MRI‐based ACSA measurements are not interchangeable.

## 1 Introduction

Anatomical muscle cross‐sectional area (ACSA) has been acknowledged as a key and suitable parameter of muscle function (Ikai and Fukunaga 1968; Maughan et al. 1983; Kanehisa et al. 1994; Blazevich et al. 2009; Moss et al. 1997). More specifically, for arm and leg muscles, ACSA has been shown to be related to joint torque generation capacity (Ikai and Fukunaga 1968; Maughan et al. 1983; Kanehisa et al. 1994; Blazevich et al. 2009). On that basis, ACSA has been used as an outcome index for the follow‐up of training (Moss et al. 1997; Narici et al. 1989, 1996), detraining (Scott et al. 2017) and postclinical interventions (Stevens et al. 2006). To measure muscle ACSA, several imaging techniques can be used, including computed tomography (CT) (Maughan et al. 1983; Moss et al. 1997; Noorkoiv et al. 2010), magnetic resonance imaging (MRI) (Blazevich et al. 2009; Narici et al. 1996; Scott et al. 2017) and ultrasonography (US) (Ikai and Fukunaga 1968; Kanehisa et al. 1994; Scott et al. 2017). Generally speaking, CT and MRI scanners are less available than US devices. In addition, US devices are much less expensive and can be handled more easily and require much less examination time. Specifically, the 2D field of view of a single US image is considered limited compared to CT and MRI (Reeves et al. 2004; Lixandrão et al. 2014), and the extended‐field‐of‐view (EFOV) technique is relevant to measure ACSA in a large field of view (Scott et al. 2017; Noorkoiv et al. 2010; Ahtiainen et al. 2010; Scott et al. 2012; Melvin et al. 2014; Rosenberg et al. 2014; Palmer et al. 2015; Franchi et al. 2020; Kwan et al. 2020; Kositsky et al. 2020; Sahinis et al. 2020; Kellis et al. 2021; Valera‐Calero et al. 2021; Sobolewski et al. 2021; Hernández‐Belmonte et al. 2022; Van den Broeck et al. 2023; Buffet‐García et al. 2024). In this line, EFOV US demonstrated high accuracy (sensitivity and specificity) in detecting quadriceps atrophy and hypertrophy (± 10 cm^2^) as well as gastrocnemius atrophy (−6 to 0 cm^2^), but not its hypertrophy (0 to + 2 cm^2^) compared to the MRI (Scott et al. 2017). These absolute changes clearly fall within the range of (8%–34%) US‐ACSA increases reported within the quadriceps and the hamstring muscle groups after concentric training (Housh et al. 1992). Therefore, ACSA measurement using US could then be considered the method of choice as long as the corresponding validity and reliability are comparable to those obtained with gold standard methods such as CT or MRI (Noorkoiv et al. 2010; Ahtiainen et al. 2010; Franchi et al. 2020; Valera‐Calero et al. 2021).

Reliability reflects the random error in repeated measurements, whereas validity illustrates the systematic error between a given method and a gold‐standard method (Bartlett and Frost 2008). Many studies on lower limb muscles have demonstrated that the EFOV‐US assessment of ACSA is generally highly reliable (Noorkoiv et al. 2010; Ahtiainen et al. 2010; Scott et al. 2012; Melvin et al. 2014; Rosenberg et al. 2014; Palmer et al. 2015; Tomko et al. 2018; Franchi et al. 2020; Kwan et al. 2020; Kositsky et al. 2020; Sahinis et al. 2020; Kellis et al. 2021; Sobolewski et al. 2021; Valera‐Calero et al. 2021; Hernández‐Belmonte et al. 2022; Van den Broeck et al. 2023), but it still differs from the MRI‐ACSA values. Much of the current literature reports underestimated ACSA values for the quadriceps (Ahtiainen et al. 2010; Scott et al. 2012), hamstrings (Kositsky et al. 2020), gastrocnemii (Scott et al. 2012) and tibialis anterior (Sponbeck et al. 2021) when using EFOV‐US compared to MRI‐ACSA values. By contrast, one study (Franchi et al. 2020) reported overestimated ACSA values in certain regions of interest for two out of the four hamstring muscles. Notably, very few studies have addressed (Franchi et al. 2020; Kositsky et al. 2020; Kellis et al. 2021) or tested (Scott et al. 2012) whether muscle size influences the validity and reliability of ACSA measurements and the conclusions drawn differ. For example, two studies on the hamstring muscles (Franchi et al. 2020; Kositsky et al. 2020) found great CSA agreement between the two imaging techniques when the CSA was largest. However, another study (Scott et al. 2012) reported the opposite when comparing the quadriceps and gastrocnemii muscles, with greater discrepancies found for the vastus lateralis.

Regarding the underlying influential factors, in the EFOV processing, large muscles need to stitch multiple images, which requires merging multiple sections of individual images to generate a final image (Kröger et al. 1998; Fornage et al. 2000). Depending on the studies, this process is considered to result either in the underestimation of US‐ACSA values (Scott et al. 2012) or in a better agreement (Franchi et al. 2020). Illustrating this discrepancy, the poorest agreement has been reported either for the largest quadriceps muscle (vastus lateralis) (Scott et al. 2012) or for small hamstring muscle (BFsh) (Franchi et al. 2020). This suggests the influence of other factors: the muscle surface curvature could affect the reliability and validity of EFOV‐US measurements (Noorkoiv et al. 2010; Fornage et al. 2000). When assessing small muscles, part of the discrepancy is likely to be influenced by the features of each imaging technique (e.g., image resolution and image depth). Considering the acknowledged lower muscle mass in females (Abe et al. 2003), it would be interesting to assess the effects of sex on the reliability and validity of EFOV‐US. Although similar reliability has been reported for the biceps femoris long head (BFlh), semitendinosus (ST) and semimembranosus (SM) muscles between males and females (Palmer et al. 2015), the effect of sex, especially on validity, has been very scarcely investigated in the lower limb muscles. Thus, a comprehensive study is still needed to assess the interchangeability of US‐ and MRI‐based ACSA measurements on a larger panel of lower limb muscles (Ahtiainen et al. 2010; Scott et al. 2012; Franchi et al. 2020; Kositsky et al. 2020; Sponbeck et al. 2021), particularly in females.

The present study aimed to assess the reliability and validity of US‐based ACSA measurements in ten lower limb muscles of different sizes and between males and females, using MRI‐based ACSA measurements as the gold standard method. Based on previous research, we hypothesized that (1) US‐ACSA measurements of lower limb muscles performed with the EFOV method would be highly reliable, that is with error values much lower than changes commonly reported in interventional studies; (2) although US‐ACSA would show strong relative agreement with MRI‐ACSA, absolute agreement would be limited due to a systematic underestimation by ultrasound; and (3) the extent of this underestimation would be influenced by muscle size and sex, with a greater bias in larger muscles and in male participants.

## 2 Materials and Methods

### 2.1 Participants

Twenty‐four healthy subjects (12 males: 26.3 ± 8.4 years, 176.8 ± 6.2 cm, and 69.6 ± 6.8 kg and 12 females: 24.8 ± 3.2 years, 165.8 ± 4.5 cm, and 59.7 ± 6.4 kg) volunteered to take part in the study after signing an informed consent form. They were moderately active, that is undertaking one to three exercise sessions of exercise per week. The study was conducted from 18 October 2021 to 11 February 2022. ACSA was measured once for each subject using both ultrasound (US) and MRI, and twice one day apart for a subgroup of 16 participants (8 women). The reliability was tested with one day in between to evaluate the short‐term consistency of image acquisition and segmentation procedures. Two post hoc analyses were conducted in order to assess whether the sample size was adequate for the study objectives. In line with previous studies (Ahtiainen et al. 2010; Rosenberg et al. 2014; Franchi et al. 2020; Walter et al. 1998), the first analysis was related to the reliability of ultrasound measurements (i.e., ICC), whereas the second analysed the agreement between MRI and ultrasound measurements. Subjects had not sustained injury in the previous 6 months and did not have a skeletal muscle disease. The project was approved by the French Research Ethic Committee (CPP IDF VII, Ethics Committee Agreement # 2021‐A00355‐36), and the written informed consent was obtained from each participant in accordance with the Helsinki Convention.

### 2.2 Muscles of Interest and Standardisation of Measurements

To measure the ACSA in ten muscles of the dominant lower limb and to avoid issue related to time constraints, participant compliance and operator burden issues, the commonly used single‐slice ACSA imaging technique, which is used in both clinical and applied research settings (Damas et al. 2016; Michel et al. 2025), was employed. Measurement positions were standardised according to the length of the femur and fibula, with 0% corresponding to their proximal end. Femur length was defined as the distance from the tip of the greater trochanter to the lateral femoral epicondyle, and leg length as the distance from the lateral fibular epicondyle to the apex of the lateral malleolus. As previously described (Franchi et al. 2020; Kositsky et al. 2020; Sponbeck et al. 2021; Balshaw et al. 2021), ACSA of each muscle was measured near the muscle belly in the following positions: RF at 30%; VL, ST and BFlh at 50% and VM at 70% of the femur length. ACSA of the gastrocnemius lateralis (GL) muscle was measured at 20% of the leg length while gastrocnemius medialis (GM) and tibialis anterior (TA) were measured at 30%. Due to limited measurement time, SM and biceps femoris short head (BFsh) were also measured at 50%. The US‐ACSA measurement sites were marked precisely on the skin with a permanent marker. For the MRI scan, corresponding bony landmarks were used to take measurements in the axial plane.

### 2.3 Ultrasound Imaging Acquisition and Analyses

As experience and training have been reported to affect the validity of US‐ACSA measurements (Franchi et al. 2020; Sobolewski et al. 2021), we decided to have a single expert with over 10 years of experience in musculoskeletal US imaging perform the entire set of US measurements. This was done to avoid the well‐recognised interoperator variability (Franchi et al. 2020; Hernández‐Belmonte et al. 2022) and focus on sources of error attributable to the measurement technique and anatomical factors. Participants were asked to lie in the prone position for the calf and hamstring measurements and in the supine position for the quadriceps and tibialis anterior measurements. They were instructed to relax during the process. The ankle joint was positioned at 15°–20° of plantar flexion on a footrest (0°: foot perpendicular to the tibia), and the knee at 175°–180° of extension (180°: knee fully extended). This position was chosen to avoid passive tension in the lower limb muscles (Kay and Blazevich 2009). ACSAs of all muscles were computed using a B‐mode US imaging device (Aixplorer Mach 30 and Linear SL18‐5, Supersonic Imagine, Aix‐en‐Provence, France) in the EFOV scan mode, except for muscles small enough to be captured in a single image. To ensure that repeated measurements were taken at the same location, body landmarks and skin labels were used in conjunction with a custom‐made positioning guide and a plastic probe holder. First, an ultrasound device was used to identify the bone landmarks (the tip of the greater trochanter, the lateral femoral epicondyle, the lateral fibular epicondyle and the lateral malleolus), and marks were made on the skin with a permanent marker. Then, the ACSA measurement locations on the thigh and lower leg were determined and marked with a permanent marker: 30%, 50% and 70% of the femur length and 20% and 30% of the leg length. A custom‐made positioning guide (a semicircular plastic cover) was used to mark horizontal lines parallel to the long axis of both thigh and lower leg at the measurement positions using a permanent marker. Next, the ultrasound probe, fitted within a 3D‐printed plastic probe holder to prevent tilting during measurement process, was slowly and carefully moved along the region of interest. A water‐based gel was positioned between the skin and the transducer to ensure acoustic contact and to counteract the effects of changes in transducer pressure. Before each ultrasound measurement, marks and lines were made with a permanent marker to ensure consistent measurement at the same location. A minimum of three successful US scans were obtained for each of the ten selected muscles during each session.

**FIGURE 1:**
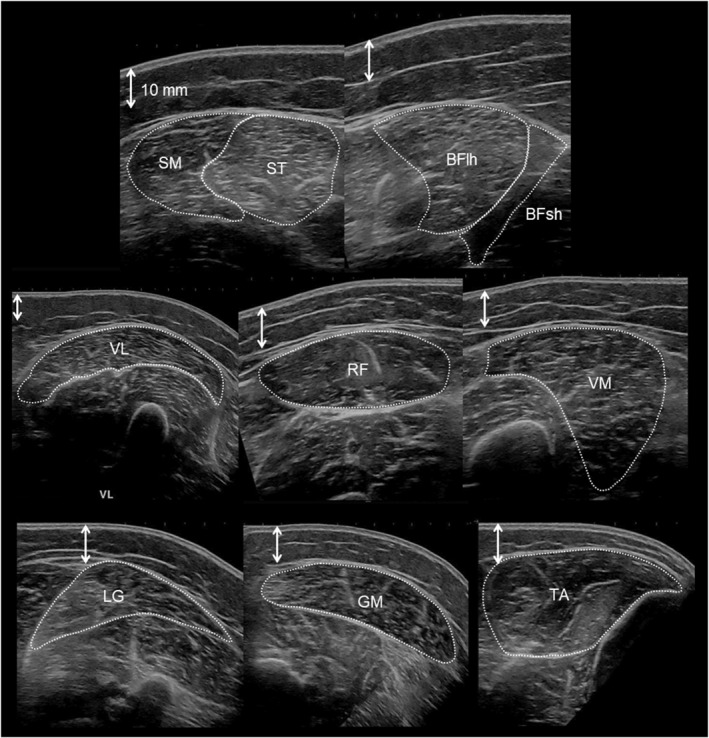
Representative EFOV ultrasound images (female participant). BFlh, biceps femoris long head; BFsh, biceps femoris short head; GL, gastrocnemius lateralis; GM, gastrocnemius medialis; RF, rectus femoris; SM, semimembranosus; ST, semitendinosus; TA, tibialis anterior; VL, vastus lateralis; VM, vastus medialis.

The in‐plane spatial resolution of the B‐mode ultrasound images was 0.12 mm^2^ per pixel. The entire set of US images was manually segmented by the same observer. Each ACSA value was quantified three times using ImageJ (NIH, Bethesda, Maryland, USA). Muscles were outlined within the visible muscle fascia (Figure 1). In cases where ultrasound images presented less visible boundaries, we referred to the corresponding B‐mode video recordings to confirm and accurately segmentation of the muscle outlines. In a few participants (mostly males), the VL and MG were too large to fit within a single EFOV image. In these cases, two separate EFOV images were taken: one medial and one lateral. Prior to segmentation, these two images were merged into a single image according to the tape marks that had been placed on the skin. The average of the three measured and calculated ACSA values was used as the representative US‐ACSA value.

### 2.4 MRI Acquisition and Analyses

Participants were positioned supine in a 3.0‐T clinical whole‐body scanner (Vida, software version XA20A, Siemens Healthineers, Erlangen, Germany). Similar to the US measurements, participants were positioned on a footrest at 15°–20° of plantar flexion (0°, foot perpendicular to the tibia) and 175°–180° of knee extension (180°, knee fully extended) to ensure a fully relaxed position. Acquisitions were performed with flexible coils positioned on the top of the lower limb and spinal coils integrated into the scanner bed on the bottom. MRI images were recorded in the transverse plane. Localized B0 shimming was performed with second‐order shims using the interactive shim programme of the manufacturer. The initial localisation scan was performed to identify the bone landmarks, that is, the greater trochanter for the upper thigh and the lateral tibial condyle for the upper leg. T1‐weighted images were acquired from two stacks (thigh and leg) using a Turbo Spin Echo sequence (TR/TE = 623/10 ms). The region of interest (ROI) covered a total of 225 mm for each stack with an in‐plane resolution of 0.7 mm^2^. The total acquisition time was 280 s.

**FIGURE 2:**
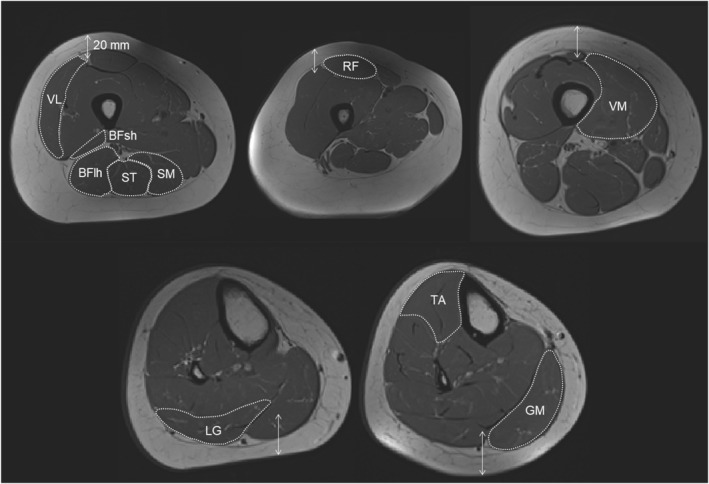
Representative MR images (female participant). BFlh, biceps femoris long head; BFsh, biceps femoris short head; GL, gastrocnemius lateralis; GM, gastrocnemius medialis; RF, rectus femoris; SM, semimembranosus; ST, semitendinosus; TA, tibialis anterior; VL, vastus lateralis; VM, vastus medialis.

ROIs for each individual muscle were manually outlined within the visible muscle fascia on T_1_‐weighted images using FSLeyes (FSL version 0.34.2, FMRIB Software Library, Oxford, UK) (Jenkinson et al. 2012) (Figure 2). Individual muscles were manually delineated in one out of every three to five slices, and the corresponding masks were automatically propagated to the remaining slices as previously reported (Ogier et al. 2017; Fouré et al. 2018). During the manual delineation process, care was taken to ensure that muscle fascia was not included. The total number of pixels was counted for each mask, and the corresponding ACSA was computed using the voxel size.

### 2.5 Statistics

Statistical analyses were performed using Statistical Analysis Software SPSS (SPSS Statistics 25, IBM Japan, Japan) and R (v4.3.2, for Windows with RStudio version 2024.04.01, R Foundation for Statistical Computing, Austria). US‐ACSA and MRI‐ACSA data were expressed as mean ± SD. Normality distribution was tested using the Shapiro–Wilk test.

*Reliability* of US‐ACSA and MRI‐ACSA measurements (Bartlett and Frost 2008) were assessed from measurements performed at one day interval. Standard errors of measurements (SEM), minimal detectable change (MDC) and intraclass correlation coefficients (*ICC*_2,__*3*_) were computed. From the ICC values, the interday reliability was classified as good (0.75–0.90) or excellent (> 0.9), according to the classification proposed by Koo and Li (Koo and Li 2016).

SEM and MDC were computed in absolute terms as follows:

SEM = SD_diff_/√2, where SD_diff_ is the standard deviation (SD) of the difference between test and retest.

$$\text{MDC}=1.96\times \sqrt{2}\times \text{SEM}$$

SEM and MDC were also computed in relative terms, that is, expressed as a percentage of the means of the first test.

*Validity* of US‐ACSA measurements (Bartlett and Frost 2008) was assessed using MRI as the reference method. To evaluate the relationship and agreement between EFOV‐US and MRI measurements, both Pearson's correlation coefficients and linear regression analyses were conducted. Pearson's correlation coefficients were classified as very strong (0.9–1), strong (0.70–0.89), moderate (0.50–0.69), low (0.30–0.49) and very low (0.0–0.29) (Mukaka 2012). A potential sex effect was assessed using ANCOVA analysis. In addition, modified Bland–Altman analysis (Bland and Altman 1986) was performed by plotting the relative differences (rDIFF (%)) between the US‐ and MRI‐ACSA values against the MRI‐CSA values. The limits of agreement were computed as the mean ± 1.96 SD.

rDIFF(%)=((US−ACSA)–(MRI−ACSA))/(MRI−ACSA)∗100

*To assess the effects of sex and muscle size on the validity of US measurements*, a linear model was used with sex and muscle size (MRI‐ACSA) as fixed factors and rDIFF (%) as the dependent variable. Then, an ANOVA analysis was performed on the model, with degrees of freedom estimated using the Satterthwaite formula.

Statistical significance was considered at *p* values < 0.05.

## 3 Results

### 3.1 Reliability of ACSA Measurements

For both US‐ and MRI‐ACSA measurements, ICC values computed for the entire set of muscles indicated an excellent reliability (ICC > 0.952 for MRI‐ACSA and ICC > 0.976 for US‐ACSA). SEM values of the US measurements were systematically lower than 2% for both sexes and for the entire set of muscles, whereas using MRI, they were slightly higher (up to 3%–4%) for SM and BFsh muscles (Tables 1 and 2). MDC values of US‐CSA and MRI‐CSA averaged 3.3 ± 0.9% and 5.1 ± 3.6% in males and 3.1 ± 1.2% and 5.0 ± 2.2% in females, for the entire set of muscles (Tables 1 and 2).

**TABLE 1:** Reliability of ACSA measurements using US (US‐ACSA) and MRI (MRI‐ACSA) in males.

Muscle	MRI‐CSA					US‐CSA				
	ICC_2,3_	SEM (cm^2^)	SEM (%)	MDC (cm^2^)	MDC (%)	ICC_2,3_	SEM (cm^2^)	SEM (%)	MDC (cm^2^)	MDC (%)
VM	0.952	0.39	1.31	1.07	3.63	0.997	0.21	0.93	0.58	2.59
VL	0.995	0.31	1.07	0.86	2.97	0.996	0.26	1.13	0.71	3.13
GM	0.991	0.15	1.05	0.40	2.92	0.976	0.24	1.93	0.65	5.36
RF	0.998	0.16	1.08	0.43	3.00	0.997	0.15	1.25	0.40	3.47
BFlh	0.995	0.12	0.90	0.33	2.51	0.998	0.09	0.79	0.24	2.19
ST	0.993	0.17	1.64	0.47	4.55	0.995	0.08	0.95	0.23	2.63
GL	0.995	0.14	1.35	0.40	3.75	0.996	0.09	1.08	0.25	2.98
SM	0.982	0.32	3.58	0.88	9.93	0.998	0.10	1.32	0.27	3.65
TA	0.982	0.15	1.80	0.41	4.98	0.986	0.08	1.19	0.23	3.29
BFsh	0.979	0.12	4.76	0.32	13.19	0.998	0.03	1.29	0.08	3.57
Average	0.986	0.20	1.86	0.56	5.14	0.994	0.13	1.19	0.36	3.29
SD	0.014	0.10	1.28	0.27	3.55	0.007	0.08	0.31	0.21	0.87

*Note:* Intraclass correlation coefficient (ICC), standard error of measurement (SEM) and minimal detectable change (MDC) are expressed as absolute values and as a percentage of the mean. Muscles are ranked by mean ACSA (see Table 3).

Abbreviations: BFlh, biceps femoris long head; BFsh, biceps femoris short head; GL, gastrocnemius lateralis; GM, gastrocnemius medialis; RF, rectus femoris, SM, semimembranosus; ST, semitendinosus; TA, tibialis anterior; VL, vastus laterals; VM, vastus medialis.

**TABLE 2:** Reliability of ACSA measurements using US (US‐ACSA) and MRI (MRI‐ACSA) in females.

	MRI‐CSA					US‐CSA				
Muscle	ICC_2,3_	SEM (cm^2^)	SEM (%)	MDC (cm^2^)	MDC (%)	ICC_2,3_	SEM (cm^2^)	SEM (%)	MDC (cm^2^)	MDC (%)
VM	0.997	0.24	1.24	0.68	3.43	0.998	0.12	0.80	0.33	2.29
VL	0.995	0.30	1.54	0.82	4.28	0.999	0.07	0.50	0.19	1.25
GM	0.998	0.22	1.69	0.60	4.68	0.999	0.09	0.80	0.25	2.30
RF	0.986	0.13	1.26	0.35	3.48	0.994	0.10	1.20	0.29	3.36
BFlh	0.995	0.15	1.60	0.43	4.44	0.997	0.08	1.00	0.21	2.65
ST	0.998	0.12	1.43	0.33	3.95	0.997	0.13	1.70	0.35	4.59
GL	0.994	0.13	1.67	0.36	4.62	0.996	0.08	1.25	0.23	3.46
SM	0.979	0.27	3.38	0.74	9.38	0.997	0.13	1.90	0.36	5.22
TA	0.988	0.08	1.16	0.22	3.21	0.992	0.05	0.80	0.13	2.21
BFsh	0.958	0.06	3.16	0.16	8.76	0.997	0.03	1.40	0.08	3.84
Average	0.989	0.17	1.81	0.47	5.02	0.997	0.09	1.13	0.24	3.12
SD	0.012	0.08	0.79	0.23	2.20	0.002	0.03	0.44	0.09	1.21

*Note:* Intraclass correlation coefficient (ICC), standard error of measurement (SEM) and minimal detectable change (MDC) are expressed as absolute values and as a percentage of the mean. Muscles are ranked by mean ACSA (see Table 3).

Abbreviations: BFlh, biceps femoris long head; BFsh, biceps femoris short head; GL, gastrocnemius lateralis; GM, gastrocnemius medialis; RF, rectus femoris, SM, semimembranosus; ST, semitendinosus; and TA, tibialis anterior; VL, vastus laterals; VM, vastus medialis.

### 3.2 Validity of US‐ACSA Measurements

**FIGURE 3:**
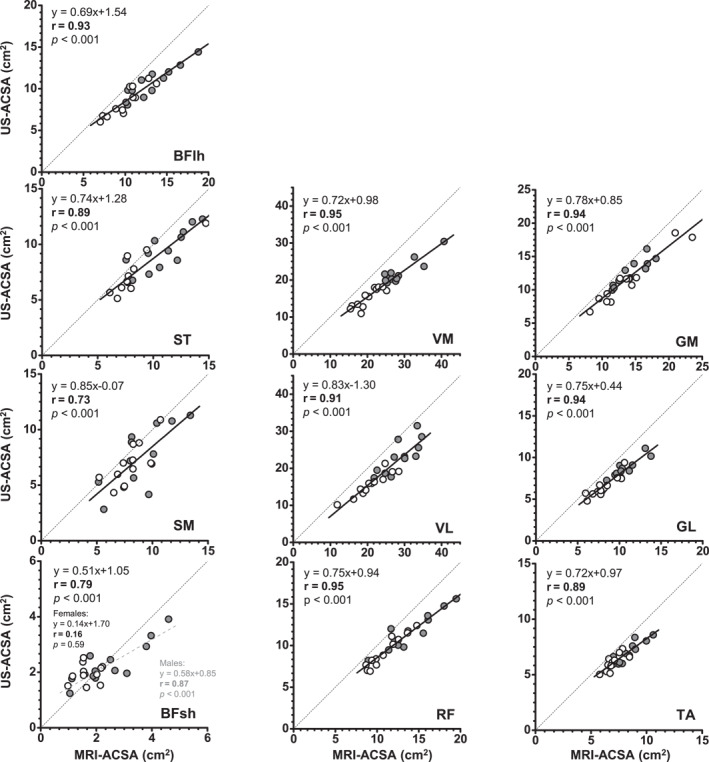
Pearson correlations between MRI‐ and US‐CSA values. The black dashed lines represent the lines of perfect concordance. Only a sex interaction was found for the BFsh muscle. To improve clarity, data for males and females are shown separately in each plot (grey filled circles: males and open circles: females). The black thick lines represent the regression lines for all participants. The grey dashed line represents the regression line for males. BFlh, biceps femoris long head; BFsh, biceps femoris short head; GL, gastrocnemius lateralis; GM, gastrocnemius medialis; RF, rectus femoris; SM, semimembranosus; ST, semitendinosus; TA, tibialis anterior; VL, vastus lateralis; VM, vastus medialis.

For most of the muscles, the regression lines did not differ significantly between the sexes. Pearson's product‐moment correlation analysis conducted between US‐ and MRI‐ACSA values indicated a sex effect for the BFsh muscle. A linear relationship was found for BFsh in males (*r* = 0.87 and *p* < 0.001), but not in females (*r* = 0.16 and *p* = 0.59) (Figure 3). Correlation analysis was performed with pooled data, regardless of sex. Very strong correlations (*r* = 0.91–0.95) were found for the quadriceps, the gastrocnemii and the BFlh muscles, whereas strong correlations (*r* = 0.73–0.89) were found for the other hamstring muscles (SM and ST) and the TA (Figure 3).

**FIGURE 4:**
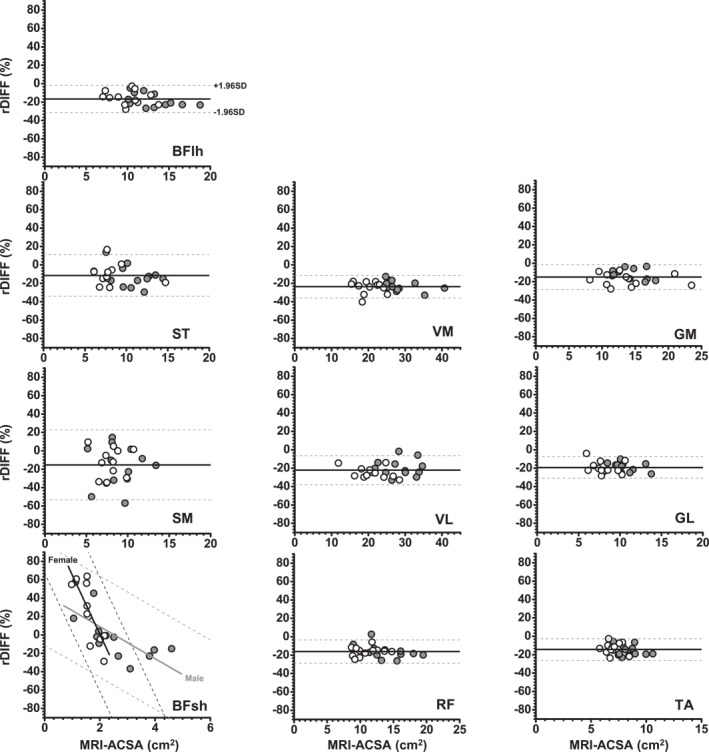
Bland–Altman plots of the relative difference (rDIFF) between MRI‐ and US‐ACSA versus CSA measured by MRI (gold standard method) for each muscle. The thick black line and dashed black line represent the mean bias and the limits of agreement, respectively. For clarity, the data for males and females are distinguished in each plot (grey filled circles: males and open circles: females). For BFsh, the grey and black lines represent males and females, respectively. BFlh, biceps femoris long head; BFsh, biceps femoris short head; GL, gastrocnemius lateralis; GM, gastrocnemius medialis; RF, rectus femoris; SM, semimembranosus; ST, semitendinosus; TA, tibialis anterior; VL, vastus lateralis; VM, vastus medialis.

Bland–Altman analysis (Table 3 and Figure 4) showed systematic differences between the MRI‐ and US‐ACSA measurements. US‐ACSA measurements were consistently lower than MRI measurements, with corresponding biases ranging from −11.5% to −23.7%. The limits of agreement for all muscles were 72.5% (upper limit) and −53.4% (lower limit). A positive bias was found only for the BFsh muscle in females (21.9%). Linear model analysis showed that muscle size (MRI‐ACSA) and sex had no effect on rDIFF (%), except for the BFsh. A significant interaction between muscle size (MRI‐ACSA) and sex was found for this muscle (*p* < 0.01), with a steeper negative correlation between muscle size and rDIFF (%) observed in females (slope coefficient = −64.4, *r* = −0.80, and *p* < 0.05) compared to males (slope coefficient = −17.4, *r* = −0.70, and *p* < 0.01).

**TABLE 3:** MRI‐ and US‐ACSA values, along with corresponding relative bias and limits of agreement.

Muscle	MRI‐ACSA (cm^2^)	US‐ACSA (cm^2^)	Bias (cm^2^) [%]	Upper LoA [%]	Lower LoA [%]
VM	24.22 ± 6.00	18.67 ± 4.56	−5.85 [−23.7%]	−11.4%	−36.0%
VL	24.93 ± 5.96	19.44 ± 5.43	−5.49 [−22.2%]	−6.4%	−39.1%
GM	14.04 ± 3.51	11.90 ± 2.93	−2.14 [−15.0%]	−1.5%	−31.1%
RF	12.51 ± 2.98	10.45 ± 2.38	−2.06 [−16.1%]	−3.5%	−28.7%
BFlh	11.59 ± 2.85	9.58 ± 2.12	−2.01 [−16.7%]	−1.9%	−31.4%
ST	9.56 ± 2.62	8.38 ± 2.17	−1.18 [−11.5%]	11.2%	−34.1%
GL	9.29 ± 2.00	7.48 ± 1.61	−1.81 [−19.3%]	−7.5%	−31.1%
SM	8.49 ± 2.00	7.18 ± 2.33	−1.30 [−15.3%]	22.7%	−53.4%
TA	7.72 ± 1.16	6.59 ± 0.95	−1.13 [−14.4%]	−2.6%	−26.3%
BFsh	2.10 ± 0.94	2.14 ± 0.60	0.03 [10.8%]	72.5%	−50.8%

*Note:* Values are reported as the mean ± SD and are ranked by mean ACSA. Negative values indicate that the US‐ACSA value was smaller than the corresponding MRI‐ACSA value.

Abbreviations: BFlh, biceps femoris long head; BFsh, biceps femoris short head; GL, gastrocnemius lateralis; GM, gastrocnemius medialis; RF, rectus femoris, SM, semimembranosus; ST, semitendinosus; TA, tibialis anterior; VL, vastus laterals; VM, vastus medialis.

## 4 Discussion

In the present study, we assessed the reliability and validity of US‐based ACSA measurements for 10 lower limb muscles in both males and females. We used the EFOV method and considered MRI as the gold standard method. Supporting our first hypothesis, US‐ACSA measurements of lower limb muscles performed using EFOV were highly reliable, with error values much lower than changes commonly reported in interventional studies. Partly supporting our second hypothesis, a strong correlation was found between US‐ and MRI‐ACSA values as well as consistent ASCA underestimation by the US technique. Rejecting the third hypothesis, the validity of US‐ACSA values was unaffected by muscle size or sex across the entire set of muscles, with the exception of the BFsh.

As indicated by the high ICC variables, US‐ACSA measurements demonstrated excellent reliability for the whole set of muscles and for both sexes. This finding is consistent with previous studies that did not test the effect of sex on the hamstrings (Franchi et al. 2020; Kositsky et al. 2020; Kellis et al. 2021), quadriceps (Ahtiainen et al. 2010; Scott et al. 2012; Melvin et al. 2014; Kwan et al. 2020; Sahinis et al. 2020; Kellis et al. 2021; Sobolewski et al. 2021; Van den Broeck et al. 2023; Buffet‐García et al. 2024; Tomko et al. 2018), gastrocnemii (Scott et al. 2012; Rosenberg et al. 2014; Van den Broeck et al. 2023) and TA muscles (Sponbeck et al. 2021; Van den Broeck et al. 2023), and with the study of (Palmer et al. 2015) on the hamstring muscles, which tested the effect of sex. The high reliability of US‐ACSA measurements is further supported by the low SEM and MDC values found across most muscles. The SEM values for US‐ACSA measurements were 1.19% (± 0.31) and 1.13% (± 0.44) for males and females, respectively. Those for MRI‐ACSA were 1.86% (± 1.28) and 1.81% (± 0.79), respectively. As expected, these values are much lower than the changes in ACSA reported following exercise interventions. For instance, using either US or MRI, an increase of 8%–13% in ACSA has been reported following resistance training (Narici et al. 1989; Ahtiainen et al. 2010), whereas a 4%–10% increase has been reported immediately and 24–96 h after repeated eccentric muscle actions (Takahashi et al. 1994; Macchi et al. 2024). Unexpectedly, the SEM (%) for MRI were higher than the corresponding US‐based values for most muscles. In particular, the MRI‐ACSA values for two of the hamstring muscles (BFsh and SM) showed the highest SEM values, approaching 5%. These larger SEMs values can primarily be attributed to differences in image resolution affecting fascia visibility. More specifically, the spatial (in‐plane) resolution of our ultrasound images was estimated to be approximately 0.12 mm^2^/pixel. In contrast, the in‐plane resolution of the MRI images was 0.7 mm^2^. This lower MRI resolution made it more difficult to clearly identify the fascia boundaries of small or deep muscles in MRI, such as the BFsh and the SM, especially when the fascia is thin. In such cases, segmentation becomes more error‐prone, resulting in higher SEM values. Therefore, supporting our first hypothesis, the current SEM values indicate that the US‐ACSA method is accurate enough to allow training and fatigue follow‐up of lower limb muscles in future studies. However, caution should still be exercised, given that MDC values of more than 4% were found for the GM muscle of males and the ST and SM muscles of females.

Partly supporting our second hypothesis, the Pearson product‐moment analysis revealed a strong correlation between US‐ACSA and MRI‐ACSA as well as a consistent trend of ACSA underestimation by the US technique across most (but not all) muscles and sexes. Regarding the US‐ACSA validity, the major exception was the BFsh muscle in females, for which no significant correlation was found with the MRI values. This may be due to the small ACSA value of this muscle in females (lower than 2.0 cm^2^), which makes segmentation more challenging. In addition, SM had the second lowest correlation coefficient (*r* = 0.73 for all participants). These results are consistent with weaker correlations between US‐ and MRI‐ACSA values that have been reported previously for the SM and BFsh compared to the ST and BFlh hamstring muscle (Franchi et al. 2020; Kositsky et al. 2020). When comparing hamstring muscles ACSA measured at different points of femur length, (Franchi et al. 2020) reported a better agreement between both techniques at larger than at smaller ACSA sites. In the present study, this explanation may apply to the BFsh, for which the ACSA was 3–11 times smaller than that of other recorded muscles, regardless of the technique. In contrast, this explanation cannot hold for the SM muscle. Although the group mean SM ACSA was only three times larger than the BFsh ACSA, it was close to the TA ACSA (8.49 vs. 7.72 cm^2^), for which a high correlation (*r* = 0.89) was found. Other possible explanations for the lower observed validity for SM and BFsh could be their anatomical location (Figure 1) and fascia thickness. Due to the 50% measurement locations used for SM and BFsh, some parts of these muscles were deeply located, which could have made the fascia less visible in the US images (Figure 1). Furthermore, the SM and BFsh fascia were rather thin, making them less visible fascia in both MR and US images (Figures 1 and 2). Overall, the US is considered a valid method for ACSA measurements with high relative agreement with MRI for most lower limb muscles. However, it is important to keep in mind that a strong correlation between MRI and US values does not mean a substantial similarity. The Bland–Altman analysis revealed that EFOV‐US systematically underestimated ACSA compared to MRI, but with the exception of the BFsh muscle. Absolute and relative underestimation ranged from −5.85 cm^2^ for VM to −1.13 cm^2^ for TA and from −23.7% for VM to −11.5% for ST. These discrepancies are consistent with or slightly larger than the underestimation ranges reported by previous studies (Ahtiainen et al. 2010; Scott et al. 2012; Kositsky et al. 2020; Sponbeck et al. 2021), but it differs from the overestimation reported for SM and BFsh muscles (Franchi et al. 2020). These findings clearly indicate that US and MRI are not interchangeable in either interventional or cross‐sectional study designs intended to measure ACSA.

Supporting the third hypothesis, the extent of underestimation was greater for larger muscles such as the VM (−5.85 cm^2^ [−23.7%]). This finding is consistent with (Scott et al. 2012) who reported that larger muscle size of RF, VL, GM and GL among different participants resulted in a greater bias. This underestimation may be due to the difficulty of stitching multiple images together as part of the EFOV processing. This process requires several sections of individual images to be merged to generate a final image (Scott et al. 2012; Kröger et al. 1998; Fornage et al. 2000). In line with this, previous studies have reported smaller biases when US images were reconstructed manually (Reeves et al. 2004; Lixandrão et al. 2014). This can be explained by the need to assemble more images using the EFOV method for larger muscles and by the longer boundaries of these muscles eventually introducing additional errors. Overall, EFOV‐US would systematically underestimate ACSA measurements as compared to MRI and so especially in larger muscles. It should be emphasised that the maximum difference in bias between muscles was 12.2% (e.g., −23.7% in VM vs. −11.5% in SM), which is much smaller than the absolute magnitude of the systematic errors themselves. For instance, the largest bias was −23.7% in VM and the widest limits of agreement was −53.4% in SM. Both values far exceed the expected changes from typical training interventions (e.g., 8%–13% increase after resistance training) (Narici et al. 1989; Ahtiainen et al. 2010). Many potential reasons could contribute to this large systematic bias, such as muscle geometry, the US machines' EFOV algorithm, image resolution and operator‐related factors. Regarding the relative bias across participants for each muscle, there was no effect of muscle size and sex on validity (except for BFsh). This means that the relative variability was similar across US measurements and group comparisons could be performed regardless of sex. The negative correlation between muscle size (MRI‐ACSA) and rDIFF values for BFsh in both males and females suggests that the small size of this muscle is the primary cause of the large observed error. In clinical practice, as US‐ACSA and MRI‐ACSA are not interchangeable, using MRI‐based reference values for muscle CSA would prevent US‐ACSA from being used to compare a patient's value to that reference (e.g., to assess whether a muscle is atrophied).

The present study has several limitations. ACSA values were quantified three times, but only for a single slice located near the largest muscle belly, for all muscles except BFsh and SM. A single slice is commonly used in applied research settings. Future studies should assess the reliability and validity of US measurements using volumetric (3D) data along the muscle length (proximal vs. distal) (Esformes et al. 2002) rather than 2D data. Our study was conducted in healthy individuals between 20 and 45 years of age to minimise the effect of age‐related changes in muscle fat infiltration and atrophy (Engelke et al. 2022). As muscle fat content increases with age, ultrasound attenuation and scattering increase (Wijntjes and van Alfen 2021), which can make segmentation more difficult. In that context, our results may not hold for specific groups, such as individuals with high fat thickness, muscle atrophy, elderly people or strength‐trained athletes. Additionally, the potential contribution of unknown effects of probe scanning speed and the manufacturer's proprietary image‐stitching algorithm to measurement errors should be considered, but these could not be tested directly in the present study. Other potential sources of errors, such as muscle curvature, muscle depth and inter‐rater reliability, should also be evaluated in the future.

## 5 Conclusion

The present study demonstrated that US‐ACSA measurements using the EFOV method are highly reliable across a wide range of lower limb muscles, regardless of muscle size or participant sex. However, although US and MRI‐based measurements were strongly correlated for most muscles, US systematically underestimated ACSA values. Although it was expected that larger muscles would introduce greater variability, the extent of relative underestimation was not consistently associated with muscle size or sex except for the BFsh. These findings confirm that, regardless of muscle size and sex, US‐ and MRI‐ACSA measurements are not interchangeable. Nevertheless, US may be appropriate for assessing relative ACSA differences for interventional purposes and group comparisons on most lower limb muscles.

## Funding

Research supported by: Centre National de la Recherche Scientifique (10.13039/501100004794)

## Conflicts of Interest

The authors declare no conflicts of interest.

## Data Availability

The data that support the findings of this study are available from the corresponding author upon reasonable request.
